# The adipokine adiponectin has potent anti-fibrotic effects mediated via adenosine monophosphate-activated protein kinase: novel target for fibrosis therapy

**DOI:** 10.1186/ar4070

**Published:** 2012-10-23

**Authors:** Feng Fang, Lei Liu, Yang Yang, Zenshiro Tamaki, Jun Wei, Roberta G Marangoni, Swati Bhattacharyya, Ross S Summer, Boping Ye, John Varga

**Affiliations:** 1 Division of Rheumatology, Northwestern University Feinberg School of Medicine, McGaw M230, 240 E Huron Street, Chicago, IL, 60611 USA; 2 School of Life Science and Technology, China Pharmaceutical University, 24 Tongjiaxiang Street, Nanjing, Jiangsu, 210009 China; 3 Pulmonary Center, Boston University School of Medicine, 850 Harrison Avenue, Boston, MA, 02118 USA

## Abstract

**Introduction:**

Fibrosis in scleroderma is associated with collagen deposition and myofibroblast accumulation. Peroxisome proliferator activated receptor gamma (PPAR-γ), a master regulator of adipogenesis, inhibits profibrotic responses induced by transforming growth factor-ß (TGF-β), and its expression is impaired in scleroderma. The roles of adiponectin, a PPAR-γ regulated pleiotropic adipokine, in regulating the response of fibroblasts and in mediating the effects of PPAR-γ are unknown.

**Methods:**

Regulation of fibrotic gene expression and TGF-ß signaling by adiponectin and adenosine monophosphate protein-activated (AMP) kinase agonists were examined in normal fibroblasts in monolayer cultures and in three-dimensional skin equivalents. AdipoR1/2 expression on skin fibroblasts was determined by real-time quantitative PCR.

**Results:**

Adiponectin, an adipokine directly regulated by PPAR-γ, acts as a potent anti-fibrotic signal in normal and scleroderma fibroblasts that abrogates the stimulatory effects of diverse fibrotic stimuli and reduces elevated collagen gene expression in scleroderma fibroblasts. Adiponectin responses are mediated via AMP kinase, a fuel-sensing cellular enzyme that is necessary and sufficient for down-regulation of fibrotic genes by blocking canonical Smad signaling. Moreover, we demonstrate that endogenous adiponectin accounts, at least in part, for the anti-fibrotic effects exerted by ligands of PPAR-γ.

**Conclusions:**

These findings reveal a novel link between cellular energy metabolism and extracellular matrix homeostasis converging on AMP kinase. Since the levels of adiponectin as well as its receptor are impaired in scleroderma patients with progressive fibrosis, the present results suggest a potential role for defective adiponectin expression or function in progressive fibrogenesis in scleroderma and other chronic fibrosing conditions. Restoring the adiponectin signaling axis in fibroblasts might, therefore, represent a novel pharmacological approach to controlling fibrosis.

## Introduction

Scleroderma or systemic sclerosis (SSc) is a chronic autoimmune disease associated with fibrosis in multiple organs [1]. Fibrosis in the skin is due to overproduction of collagen and other extracellular matrix (ECM) components by activated fibroblasts accompanied by progressive loss of subcutaneous adipose tissue [2]. Transforming growth factor-β (TGF-β) is a key mediator of fibrosis that initiates and sustains fibroblast activation and myofibroblast differentiation [3]. A variety of cell-autonomous regulatory mechanisms exist to control fibroblast activation and prevent aberrant constitutive fibrogenesis. Peroxisome proliferator-activated receptor gamma (PPAR-γ) is a pleiotropic nuclear receptor implicated in the regulation of adipogenesis [4]. Emerging evidence also implicates PPAR-γ in ECM accumulation and connective tissue homeostasis, and natural and synthetic PPAR-γ ligands are potent inhibitors of fibrotic responses [5].

Adiponectin is a multi-functional 30 kD adipokine that regulates insulin sensitivity, energy balance and cellular metabolism [6]. The expression of adiponectin is tightly regulated by PPAR-γ, and its levels in circulation are decreased in patients with obesity, type 2 diabetes and metabolic syndrome [7]. In contrast, serum levels are raised by PPAR-γ agonist treatment in mice and in humans [8]. Significantly, recent studies demonstrate that adiponectin levels are reduced in patients with diffuse cutaneous scleroderma, and are inversely correlated with disease activity, severity and duration [9-12]. These observations point to a potential role for adiponectin in the pathogenesis of scleroderma, but the underlying mechanisms are not currently understood.

The mechanisms of action accounting for the metabolic effects of adiponectin have been extensively characterized [13,14]. Biological activity is initiated through adiponectin binding to the cell membrane receptors AdipoR1, AdipoR2 and T-cadherin. The central modulator of the adiponectin signaling cascade is AMP kinase, a key intermediate in cellular energy metabolism [15]. Binding of AMP induces AMP kinase phosphorylation and activation, which both promotes catabolic energy-producing pathways and inhibits anabolic energy-consuming pathways [16]. Whereas the importance of deregulated adiponectin and AMP kinase signaling in metabolic diseases has been long appreciated [17], AMP kinase function in the context of fibrogenesis has not been thoroughly addressed, although emerging evidence suggests that adiponectin might play a significant role. Adiponectin and AMP kinase activation inhibit hepatic stellate cell proliferation and attenuate liver fibrosis [18-20]. In other studies, adiponectin was shown to prevent cardiomyocyte hypertrophy and myocardial fibrosis [21-23].

Fibrosis in scleroderma is associated with impaired PPAR-γ expression and activity and reduced adiponectin levels, which may be a direct consequence of the PPAR-γ defect [11,12,24,25]. In light of these intriguing recent observations, we sought to gain a better understanding of the role of adiponectin in the modulation of collagen synthesis and myofibroblast differentiation in fibroblasts. Results using two-dimensional monolayer cultures and three-dimensional full-thickness human skin equivalents demonstrate that adiponectin potently suppressed the expression of Type I collagen and α-smooth muscle actin (α-SMA) in normal and scleroderma fibroblasts, and abrogated the stimulation of these responses elicited by TGF-β. The inhibitory effects of adiponectin were mediated by activation of AMP kinase. Moreover, genetic deletion of adiponectin in mouse fibroblasts abrogated the inhibition of TGF-β signaling elicited by PPAR-γ agonists. The expression of adiponectin receptor 1 was selectively reduced in skin biopsies from patients with scleroderma. Taken together, these findings indicate that the adiponectin/AMP kinase pathway may play a previously unrecognized important homeostatic role in ECM regulation, and its defective function contributes to aberrant fibroblast activation in the pathogenesis of fibrosis. The adiponectin signaling pathway, therefore, represents a novel therapeutic target in scleroderma.

## Materials and methods

### Cell culture and reagents

Primary fibroblast cultures were established by explantation from neonatal foreskin biopsies, or from skin biopsies from healthy adults and scleroderma patients obtained under the protocols approved by the Institutional Review Board at Northwestern University. All donors or their parents/legal guardians provided written informed consent. Mouse skin fibroblasts were established by explant culture from three-week-old adiponectin-null mice and wild-type littermates [26]. Fibroblasts were maintained in (D)MEM) supplemented with 10% fetal bovine serum (FBS) (Lonza, Basel, Switzerland), 50 μg/ml penicillin, and 50 μg/ml streptomycin in a humidified atmosphere of 5% CO_2 _at 37°C, and studied between passages 2 to 8 [27]. When fibroblasts reached confluence, growth media with 10% FBS or serum-free media supplemented with 0.1% BSA were added to the cultures for 24 hours prior to TGF-β2 (Peprotech, Rocky Hill, NJ, USA), or full-length adiponectin (Bio Vendor, Karasek, Czech Republic). In selected experiments, the AMP-activated protein kinase (AMPK) inhibitor Compound C (Sigma, St Louis, MO, USA) was added to the culture 60 minutes prior to adiponectin. Toxicity was determined using lactate dehydrogenase (LDH) assays according to the manufacturer's instructions (Biovision, Milpitas, CA, USA).

### Three-dimensional full-thickness human skin equivalents

Normal skin fibroblasts (3 × 10^5^) were suspended in 1.5 ml reconstitution buffer and (D)MEM. Cells were mixed with rat tail type I collagen (4 mg/ml, BD Biosciences, San Jose, CA, USA) and seeded in 12-well plates at 37°C for 48 hours to solidify the collagen plug. Epidermal keratinocytes (6 × 10^6^) were isolated from foreskin and suspended in E medium supplemented with 5 ng/ml epidermal growth factor (EGF) and seeded on the collagen plug [28,29]. Forty eight hours later, organotypic cultures were placed on a metal grid (BD Biosciences) and maintained at an air-medium interface by feeding with E medium every other day for five days. Metformin (1 mM) was added to the media for 24 hours followed by TGF-β (5 ng/ml). Following incubation for a further six days, cultures were harvested, RNA was isolated, and tissues were fixed in formalin. Paraffin-embedded sections (4 μm thickness) were examined by Picrosirius Red staining.

### Short interfering RNA-mediated knockdown and adenovirus infection

Fibroblasts were transfected with target-specific siRNA (Dharmacon, Lafayette, CO, USA) or scrambled control siRNA (10 nM). Twenty-four hours following transfection, fresh media were added to the cultures, and the incubations were continued for a further 24 hours. Knockdown efficiency was evaluated by determining endogenous mRNA levels by real-time qPCR.

### RNA isolation and real-time quantitative PCR (qPCR)

At the end of each experiment, cultures were harvested, RNA was isolated using RNeasy Plus mini kits (Qiagen, Valencia, CA, USA) and examined by real-time quantitative qPCR [30]. Experiments were repeated three times with consistent results. The primers used for qPCR are shown in Table 1.

**Table 1:** Primers used for real-time qPCR.

Gene	Primer sequences	Accession number
18S rRNA	olg1, 5'-CCCCATGAACGAGGGAATT-3' olg2, 5'-GGGACTTAATCAACGCAAGCTT-3'	NR_003286
GAPDH	olg3, 5'-CATGAGAAGTATGACAACAGCCT-3' olg4, 5'-AGTCCTTCCACGATACCAAAGT-3'	NM_002046
ACTA2 (a-SMA)	olg20, 5'-CAGGGCTGTTTTCCCATCCAT-3' olg21, 5'-GCCATGTTCTATCGGGTACTTC-3'	NM_001613
COL1A1	olg149, 5'-GCTGGTGTGATGGGATTC-3' olg150, 5'-GGGAACACCTCGCTCT-3'	NM_000088
AdipoR1	olg396, 5'-TGACTGGCTAAAGGACAACG-3' olg397, 5'-AAAAGAGAAACAGCACGAAACC-3'	NM_015999
AdipoR2	olg400, 5'-CAGCCATTATAGTCTCCCAGTG-3' olg401, 5'-CCGAGATGACATAGTGCAAGG-3'	NM_024551
Glut4	olg404, 5'-ACTGGACGAGCAACTTCATC-3' olg405, 5'-GAGGACCGCAAATAGAAGGAA-3'	NM_001042

Primer sequences were obtained using Primer Express 3.0 software.

Most primer pairs span two exons, except the intronless 18S rRNA. Primers were tested by dissociation curve analysis to assure only single amplicon.

### Microarray procedures and data analysis

Expression of AdipoR1/2 mRNA was interrogated in publicly available genome-wide expression scleroderma skin microarray datasets (GEO accession number: GSE9285) [31].

### Transient transfection assays

Fibroblasts at early confluence were transfected with [SBE]_4_-luc plasmids harboring four copies of a minimal Smad-binding element using SuperFect Transfection kit (Qiagen) as described [32]. Cultures were incubated in serum-free media containing 0.1% BSA for 24 hours, followed by TGF-β2 for a further 24 hours and harvested. Whole cell lysates were assayed for their luciferase activities using a dual-luciferase reporter assay system (Promega, Madison, WI, USA). In each experiment, Renilla luciferase pRL-TK (Promega) was cotransfected as control for transfection efficiency [33]. Transient transfection experiments were performed in triplicate and repeated at least twice with consistent results.

### Confocal immunofluorescence microscopy

Fibroblasts (1 × 10^4 ^cells/well) were seeded onto eight-well Lab-Tek II chamber glass slides (Nalge Nunc International, Naperville, IL, USA) and incubated in serum-free Eagle's minimal essential medium (EMEM) with 0.1% BSA for 24 hours. Fresh media with adiponectin (5 ug/ml) were added, and the incubations continued for a further 24 hours. At the end of the experiments, cells were fixed, permeabilized, and incubated with primary antibodies to Type I collagen at 1:500 dilution (Southern Biotech, Birmingham, AL, USA), or to α-SMA at 1:200 dilution (Sigma, St Louis, MO, USA). Cells were then washed with PBS and incubated with secondary antibodies at 1:500 dilution (Alexa Fluor 488 and 594, Invitrogen) and viewed under a Nikon C1Si confocal microscope.

### Western analysis

At the end of each experiment, fibroblasts were harvested and whole cell lysates subjected to Western analysis as described [30]. The following antibodies were used: Type I collagen (Southern Biotech), α-SMA (Sigma), and GAPDH (Zymed, San Francisco, CA, USA). Bands were visualized using ECL reagents (Pierce, Rockford, IL, USA).

### Statistical analysis

Statistical analysis was performed on Excel (Microsoft, Redmond, WA, USA) using Student t-test or analysis of variance (ANOVA). The results are shown as the means ± SEM. *P *<0.05 was considered statistically significant.

## Results

### Adiponectin inhibits collagen and alpha-smooth muscle actin gene expression

**Figure 1:**
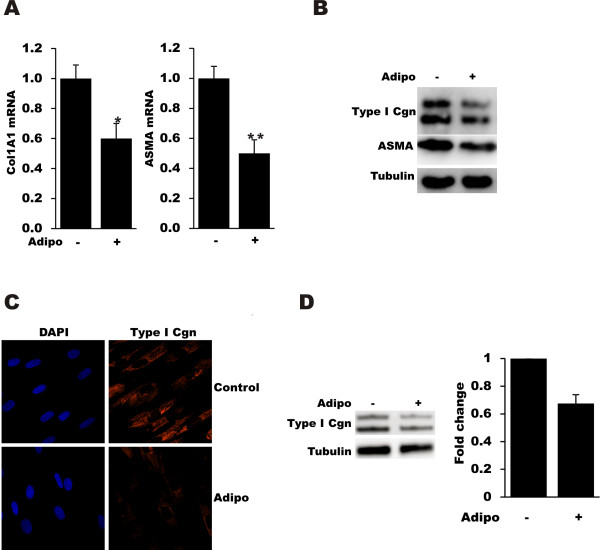
**Adiponectin inhibits collagen and alpha-smooth muscle actin gene expression**. Confluent control (**A-C**) or scleroderma (**D**) dermal fibroblasts were incubated with adiponectin (5 ug/ml) for 24 to 36 hours. **A**. Total RNA was subjected to real-time qPCR. The results represent the means ± SEM of triplicate determinations. * *P *<0.05, ** *P *<0.01. **B, D**. Whole cell lysates were analyzed by Western blot. Representative autoradiographs. **C**. Fibroblasts were immunostained with antibodies to Type I collagen (red) or α-SMA (green), or stained with DAPI (blue), and examined by confocal microscopy. Representative immunofluorescence photomicrographs. Original magnification × 60. α-SMA, α-smooth muscle actin; DAPI, 4',6-diamidino-2-phenylindole; SEM, standard error of the mean.

To investigate the regulation of fibrotic gene expression by adiponectin, foreskin fibroblasts were maintained in two-dimensional monolayer cultures. At confluence, cultures were incubated in media with an increasing concentration of adiponectin for 24 hours, and changes in gene expression were examined by real-time qPCR, Western analysis and immunocytochemistry. The results demonstrated a dose-dependent inhibition of Col1A1 and α-SMA gene expression, with a >60% reduction at 24 hours (Figure 1 and data not shown). Potent inhibition of Type I collagen and α-SMA by adiponectin was confirmed by Western analysis and immunostaining (Figure 1). Comparable results were observed in normal adult dermal fibroblasts (data not shown). Expression of both AdipoR1 and AdipoR2 mRNA in explanted fibroblasts was confirmed by real-time qPCR. Next, we investigated the effect of recombinant adiponectin in scleroderma fibroblasts. Confluent scleroderma fibroblasts (*n *= 4, Table 2) were incubated with adiponectin for 36 hours, and cell lysates were used for Western analysis. Results showed that adiponectin induced an approximately 40% decrease in collagen gene expression (Figure 1D).

**Table 2:** Clinical features of SSc patients.

Patients	Age	Sex	SSc type	Early/Late	Duration (years)	MRSS
N900	43	F				
N901	43	F				
N1003	26	M				
N1016	35	F				
S902	54	F	lcSSc	Early*	1	12
S903	39	F	dcSSc	Late	5	19
S904	33	F	dcSSc	Late	9	N/A
S1004	27	F	dcSSc	Late	10	26
S1066	21	M	dcSSc	Early	2	15
S1156	48	F	SSc/PM	Early	1	20
S1302	51	F	lcSSc	Early	2	4

*early = < 2 years from first non-Raynaud symptom at the time of skin biopsy. Fibroblasts from N900-1016 and S902-1004 were used for real-time qPCR analysis. Fibroblasts from S1004-1302 were used for Western blot analysis. MRSS, Modified Rodnan skin score; SSc, systemic sclerosis.

### Adiponectin attenuates TGF-β-induced profibrotic responses

**Figure 2:**
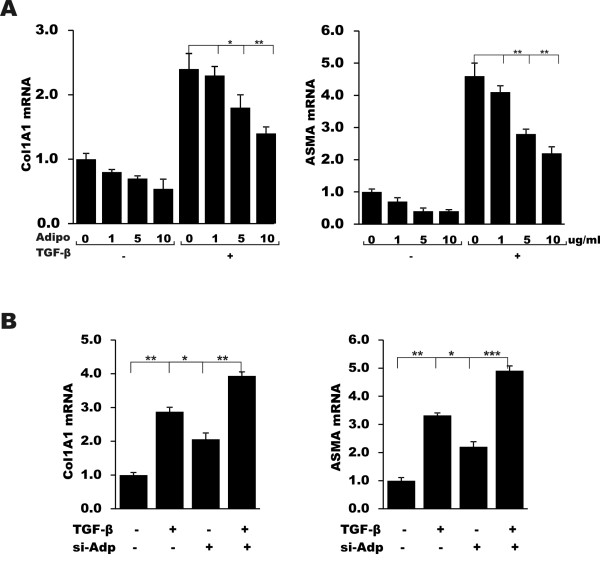
**Adiponectin attenuates TGF-β-induced profibrotic responses**. **A**. Confluent dermal fibroblasts were incubated with or without TGF-β (2 ng/ml) in the presence of the indicated concentration of adiponectin for 48 hours. Total RNA was subjected to real-time qPCR. The results represent the means ± SEM of triplicate determinations. **B**. Fibroblasts were preincubated with adiponectin-specific siRNA or control siRNA. SEM, standard error of the mean; TGF-β, transforming growth factor β

In light of the fundamental role of TGF-β in orchestrating fibrogenesis, it was of interest to evaluate how adiponectin modulated relevant responses elicited by TGF-β. For this purpose, normal fibroblasts in two-dimensional monolayer cultures were pretreated with adiponectin followed by incubation with TGF-β for a further 24 hours. The results of real-time qPCR showed that adiponectin caused a dose-dependent attenuation of collagen and α-SMA gene expression induced by TGF-β, with an almost 50% reduction at 10 μg/ml (Figure 2A). Of note, adiponectin induced an approximately four-fold increase in the levels of the TGF-β pseudoreceptor BMP and activin membrane-bound inhibitor (BAMBI), which negatively regulates TGF-β responses. To examine the possible role of endogenous adiponectin in modulating the intensity of TGF-β responses, we used an RNAi approach. The results showed that siRNA-mediated effective knockdown of adiponectin in fibroblasts significantly increased the basal levels of Type I collagen and α-SMA mRNA and protein (1.5-fold and 1.8-fold, respectively). Moreover, adiponectin-depleted fibroblasts were hypersensitive to TGF-β treatment, with significantly enhanced stimulation of collagen and α-SMA gene expression compared to fibroblasts transfected with control siRNA, suggesting an inhibitory function for endogenous adiponectin in setting the intensity of TGF-β signaling.

### Agonists of AMP kinase inhibit fibrotic gene expression and abrogate TGF-β responses

**Figure 3:**
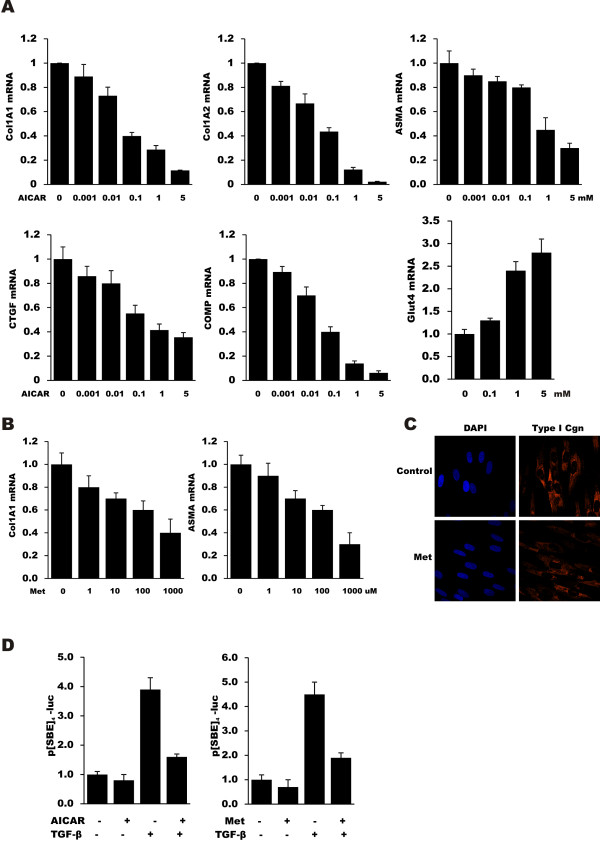
**AMP kinase agonists inhibit profibrotic gene expression**. Confluent dermal fibroblasts in two-dimensional monolayer cultures were incubated with the indicated concentrations of **(A) **AICAR or **(B) **metformin for 24 hours. Total RNA was subjected to real-time qPCR. The results represent the means ± SEM of triplicate determinations. **C**. Fibroblasts were immunostained with antibodies to Type I collagen (red), or stained with DAPI (blue). Representative immunofluorescence photomicrographs. Original magnification ×60. **D**. Fibroblasts transiently transfected with (SBE)_4_-luc were pre-treated with AICAR or metformin (1 mM) for 24 hours, followed by TGF-β (10 ng/ml) for 24 hours. Cell lysates were assayed for their luciferase activities. The results represent the means ± SEM of triplicate determinations. AICAR, 5-amino-1-β-D-ribofuranosyl-imidazole-4-carboxamide; DAPI, 4',6-diamidino-2-phenylindole; SEM, standard error of the mean; TGF-β, transforming growth factor-β.

In mesenchymal cells, adiponectin induces AMP kinase activity ([34] and data not shown). To investigate the role of AMP kinase in modulating fibrotic gene expression, fibroblasts were incubated with the selective AMP kinase agonists 5-amino-1-β-D-ribofuranosyl-imidazole-4-carboxamide (AICAR) or metformin. The results of real-time qPCR demonstrated a potent dose-dependent inhibition of Col1A1 and Col1A2 mRNA expression, with a nearly 90% reduction at 5 mM of the AMP kinase antagonists (Figure 3). There was no evidence of cellular toxicity even at the highest concentrations of AICAR or metformin tested (data not shown). In addition to collagen, multiple genes implicated in fibrogenesis showed substantial decrease in expression. To establish the specificity of the anti-fibrotic activity of AMP kinase agonists, we examined the expression of the insulin-regulated glucose transporter GLUT4, a target gene positively regulated by AMP kinase [35]. As expected, AICAR induced a substantial increase in GLUT4 mRNA expression. Both AMP kinase agonists potently attenuated the fibrotic responses induced by TGF-β (data not shown). To investigate the mechanism, transient transfection assays were performed. The results showed that adiponectin incubation resulted in disruption of canonical Smad signaling, as shown in transient transfection assays with the Smad-responsive minimal promoter (SBE)_4_-luc (Figure 3D).

**Figure 4:**
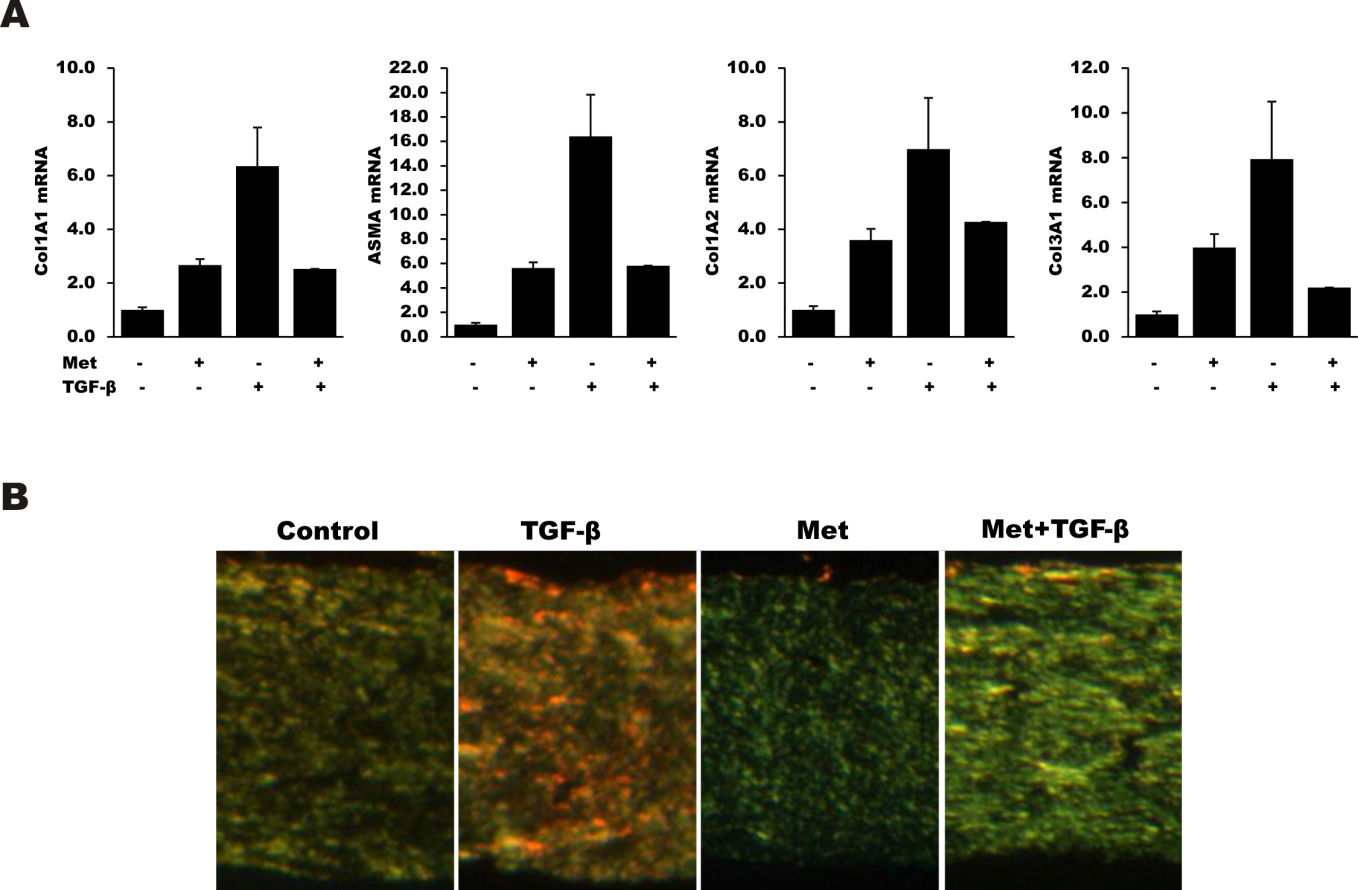
**AMP kinase agonists attenuate TGF-β-induced profibrotic responses in three-dimensional full-thickness human skin equivalents**. Fibroblasts embedded in three-dimensional full-thickness skin equivalents were incubated with metformin (1 mM) with or without TGF-β (5 ng/ml) for six days. At the end of the experiments, rafts were harvested. **A**. Real-time qPCR. The results represent the means ± SEM of triplicate determinations. **B**. 4-μm thick paraffin-embedded sections were stained with Picrosirius Red. Mature highly cross-linked collagen fibers in the dermal compartment appear red when visualized under polarized light, whereas less mature collagen fibers containing few cross-links appear yellow/green. Representative images (original magnification ×40). SEM, standard error of the mean; TGF-β, transforming growth factor-β.

The organotypic raft culture (ORC) model is a three-dimensional full-thickness human skin equivalent that is a powerful approach to studying fibroblast function in the context of fibrogenesis [28]. This full thickness human skin equivalent model allows us to examine fibroblast behavior where the biomechanical forces impacting the fibroblasts are relevant to the physiologically relevant context of skin [28]. The three-dimensional full-thickness skin equivalents were incubated with metformin (1 mM) with or without TGF-β (5 ng/ml) for six days. Results from real-time qPCR showed that while TGF-β induced a substantial increase in fibrotic gene expression, treatment with metformin abrogated the effect (Figure 4A). Picrosirius Red staining showed that TGF-β induced a notable accumulation of strongly birefringent red collagen fibers, indicating highly cross-linked collagen, in the dermal compartment. In contrast, pretreatment of the rafts with metformin prevented collagen maturation, with a predominance of green, less cross-linked collagen fibers (Figure 4B), confirming that metformin abrogated TGF-β induced collagen protein accumulation.

**Figure 5:**
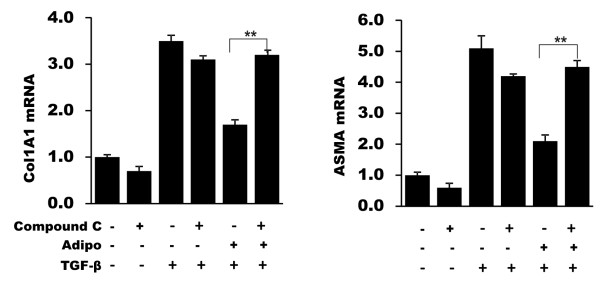
**AMP kinase mediates suppression of profibrotic responses by adiponectin**. Confluent dermal fibroblasts in two-dimensional monolayer cultures were incubated with adiponectin (5 ug/ml) in media with TGF-β (2 ng/ml) in the presence or absence of Compound C (10 uM) for 48 hours. Total RNA was harvested and subjected to real-time qPCR. The results represent the means ± SEM of triplicate determinations. SEM, standard error of the mean; TGF-β, transforming growth factor-β.

To directly examine the role of AMP kinase in mediating the antifibrotic effects of adiponectin, a chemical inhibitor of AMP kinase activity was used [36]. In fibroblasts preincubated with Compound C, a selective and potent AMP kinase inhibitor, the inhibitory effects of adiponectin on TGF-β-induced collagen and α-SMA mRNA and protein were completely abrogated (Figure 5).

### Adiponectin mediates the anti-fibrotic effects of PPAR-γ ligands

**Figure 6:**
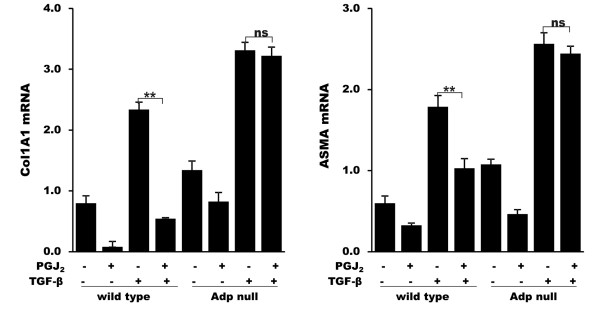
**Adiponectin mediates the anti-fibrotic effects of PPAR**-**γ ligands**. Dermal fibroblasts from adiponectin-null mice and wild-type littermates in parallel were incubated with indicated concentrations of PGJ_2_, followed by TGF-β (2 ng/ml) for 24 hours. Total RNA was subjected to real-time qPCR. The results represent the means ± SEM of triplicate determinations. PGJ_2_, prostaglandin J_2_; SEM, standard error of the mean; TGF-β, transforming growth factor-β.

We have shown previously that both pharmacological and endogenous ligands of PPAR-γ inhibited collagen gene expression, and abrogated the stimulation of fibrotic responses elicited by TGF-β [37]. Moreover, rosiglitazone, a PPAR-γ ligand inhibited the over-expression of fibrotic genes in fibroblasts explanted from scleroderma patients [24]. The anti-fibrotic activities of these ligands were blocked by the irreversible PPAR-γ antagonist GW9662, indicating that they were largely PPAR-γ-dependent [38]. Adiponectin is a direct transcriptional target of PPAR-γ, and its expression in both adipocytes and fibroblasts is tightly regulated via activated PPAR-γ binding to cognate DNA recognition sequences in the adiponectin gene promoter [11,39]. In order to investigate the potential role of endogenous adiponectin in mediating the anti-fibrotic effects of PPAR-γ ligands, we examined the effect of prostaglandin J2 (PGJ_2_) in adiponectin-null mouse skin fibroblasts. Consistent with the results using RNAi, we found that collagen and α-SMA gene expression were significantly elevated in both unstimulated and TGF-β-stimulated fibroblasts lacking adiponectin compared to wild type control fibroblasts, confirming the significant role of cellular adiponectin in modulating the intensity of TGF-β-induced fibrotic responses (Figure 6). Importantly, while PGJ_2 _elicited substantial down-regulation of TGF-β responses in wild type fibroblasts, as shown previously [25], no significant PGJ_2 _effect on the stimulatory response was seen in adiponectin-null fibroblasts.

### Adiponectin attenuates LPS-induced profibrotic responses

**Figure 7:**
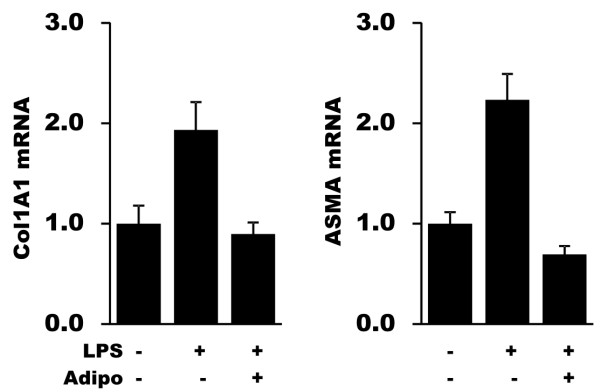
**Adiponectin attenuates LPS-induced profibrotic responses**. Confluent dermal fibroblasts were incubated with LPS (1 ug/ml) in the absence or presence of adiponectin (10 ug/ml) for 24 hours. Total RNA was subjected to real-time qPCR. The results represent the means ± SEM of triplicate determinations. LPS, lipopolysaccharide; SEM, standard error of the mean.

We next sought to determine if the anti-fibrotic effects of adiponectin were specific for TGF-β, or more generalized for other profibrotic stimuli. To this end, fibroblasts were incubated with lipopolysaccharide (LPS), a potent ligand of Toll-like receptor 4 (TLR4). LPS induced a time-dependent stimulation of collagen and αSMA gene expression in normal fibroblasts (Figure 7 and data not shown). However, pretreatment of the cultures with adiponectin completely abrogated the stimulatory effects of LPS.

### Adiponectin receptor expression in scleroderma

**Figure 8:**
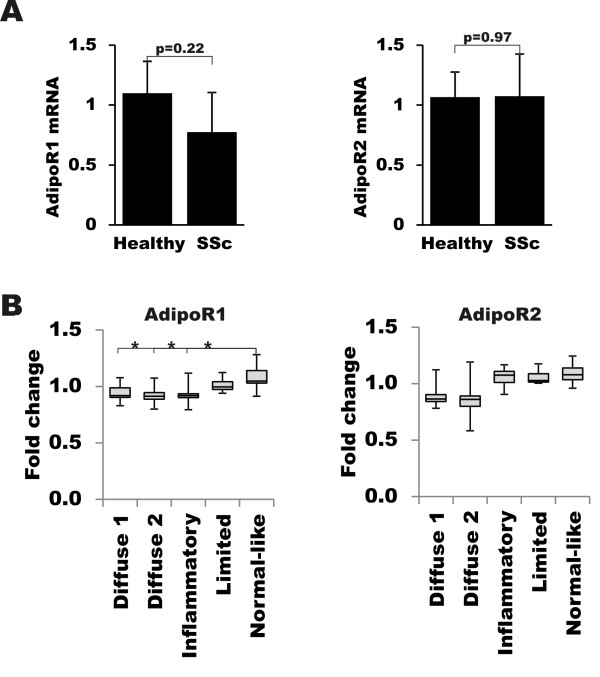
**Adiponectin receptor expression in scleroderma. A**. Total RNA was isolated from confluent fibroblasts explanted from scleroderma patient (*n *= 4) and healthy controls (*n *= 4), and subjected to real-time qPCR. The results represent the mean ± SEM of triplicate determinations. **B**. AdipoR1/2 mRNA expression was interrogated in publicly available genome-wide expression scleroderma skin biopsy microarray datasets (GEO accession number: GSE9285) [31]. Fold-change in mRNA levels was normalized with average expression levels of the samples in the entire cohort. Box plots indicate the range of lower and upper quartiles. * *P *<0.05. SEM, standard error of the mean.

Adiponectin-induced cellular responses are mediated through activation of the adiponectin receptors AdipoR1 and AdipoR2 [40]. In order to investigate the adiponectin signaling axis in scleroderma, we examined AdipoR expression. Fibroblasts were explanted from skin biopsies from the affected lesional forearm of four patients with scleroderma (Table 2), and age and sex-matched healthy controls (*n *= 4) and grown to confluence, when total RNA was isolated and subjected to real-time qPCR. The results showed approximately 40% lower levels of AdipoR1 mRNA in scleroderma fibroblasts compared to normal fibroblasts, but the differences were not statistically significant (*P *= 0.22) (Figure 8). AdipoR2 levels were comparable in scleroderma and control fibroblasts.

To evaluate AdipoR1/2 mRNA expression in scleroderma skin, the expression of these genes was interrogated in a publicly available microarray dataset examining gene expression in skin [31]. Biopsies clustering within the diffuse and inflammatory intrinsic subsets [31] showed an approximately 30% reduction in AdipoR1 (*P *<0.05), with a slight reduction in AdipoR2 (*P *= ns) expression compared to biopsies clustering with the normal-like subset (Figure 8B).

## Discussion

Persistence of activated myofibroblasts in response to chronic TGF-ß signaling underlies the progression of fibrosis in scleroderma [2]. We have demonstrated that PPAR-γ activation by endogenous ligands or pharmacological agonists exerts potent inhibitory effects on collagen gene expression and myofibroblast differentiation, and blocks TGF-ß-induced profibrotic responses, in mesenchymal cells *in vitro *[37,38]. Moreover, the PPAR-γ ligand rosiglitazone was shown to prevent and attenuate the development of dermal fibrosis in mice [41]. Significantly, recent studies have revealed a marked impairment of PPAR-γ expression and activity in skin biopsies from subsets of patients with scleroderma [25]. Moreover, explanted scleroderma fibroblasts showed reduced PPAR-γ [24]. We have previously identified a scleroderma subset with impaired PPAR-γ signaling that was associated with a strong 'TGF-ß-activated gene signature' in skin biopsies [42]. These scleroderma patients had a rather aggressive form of disease with extensive skin fibrosis. While these findings strongly implicate aberrant PPAR-γ function in the persistent fibrosis of scleroderma, the underlying molecular mechanisms remain to be elucidated.

The present studies showed that the PPAR-γ-regulated adipokine adiponectin caused a marked inhibition of collagen gene expression and myofibroblast differentiation in neonatal and normal adult skin fibroblasts as well as in scleroderma fibroblasts. Significantly, these inhibitory effects occurred at adiponectin concentrations approximating physiological plasma levels (5 to 20 μg/ml) [11,43]. Adiponectin stimulated the expression of BAMBI, an endogenous negative regulator of Smad-dependent signaling, while blocking fibrotic responses elicited by TGF-β, as well as by the TLR4 ligand LPS. While TGF-β-induced collagen production and myofibroblast transformation are known to be mediated via the canonical Smad signaling pathway [44], the mechanism underlying the fibrotic responses elicited by TLR4 ligands remain incompletely understood. A comparable antagonism between adiponectin and LPS was described in the context of LPS-dependent fibrogenesis in adventitial fibroblasts [45]. The inhibitory effects of adiponectin on fibrotic responses were associated with activation of AMP kinase, a stress-induced metabolic master switch that plays a key role in maintaining energy homeostasis. By detecting and responding to cellular nutrient and energy fluctuations, heterotrimeric AMP kinase promotes catabolic energy-producing pathways to enhance cellular glucose uptake, fatty acid oxidation, and GLUT4 biogenesis [46]. In the present studies, pharmacological AMP kinase agonists mimicked the inhibitory effect of adiponectin on profibrotic gene expression and Smad-dependent signaling, while the selective AMP kinase inhibitor Compound C rescued TGF-β stimulation of fibrotic genes in the presence of adiponectin. Moreover, transient transfection experiments indicate that AMP kinase attenuation resulted in abrogation of canonical Smad-dependent TGF-β signaling. While previous studies have highlighted the anti-inflammatory, anti-oxidant and fatty acid-regulating activities of AMP kinase [18,47], the present studies reveal important functions for adiponectin in modulating fibrogenesis. The mechanism underlying the anti-fibrotic activities of adiponectin and their significance in health and fibrosis remains to be elucidated.

Adiponectin is an adipocyte-derive pleiotropic hormone with key protective roles in diabetes and atherosclerosis [17,48,49]. Sequence-specific recognition of the adiponectin gene promoter PPRE element by activated PPAR-γ results in enhanced adiponectin transcription [7]. Recent studies expand the spectrum of the biological activities ascribed to adiponectin, including important roles in regulating inflammation and cancer [50]. Cellular adiponectin responses are mediated via the seven transmembrane domain type 1 and type 2 adiponectin receptors as well as T-cadherin [17]. Obesity is associated with reduced expression of adiponectin receptors in various tissues, contributing to a state of adiponectin resistance [51].

We and others have shown that adiponectin levels are reduced in the serum and lesional skin from patients with scleroderma [10-12]. Adiponectin levels were inversely correlated with the skin score, a measure of fibrotic skin involvement, and scleroderma patients with the most extensive skin fibrosis had the lowest adiponectin levels [11]. Moreover, patients responding to anti-fibrotic treatment with improved skin scores or lung function displayed a time-dependent increase in serum adiponectin levels [11,12].

**Figure 9:**
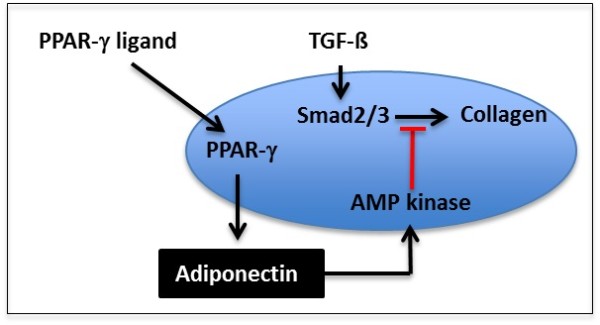
**Adiponectin suppresses fibrotic responses**. Ligands of PPAR-γ induce adiponectin which triggers autocrine or paracrine activation of AMP kinase. AMP kinase in turn blocks TGF-β/Smad signaling and consequent stimulation of profibrotic gene expression. PPAR-γ, peroxisome proliferator activated receptor gamma; TGF-β, transforming growth factor-β.

The important role for adiponectin in negative regulation of connective tissue remodeling suggested by these findings is concordant with recent observations. For instance, adiponectin was shown to down-regulate connective tissue growth factor expression in hepatocytes and hepatic stellate cells, and blocked the stimulatory effect elicited by TGF-ß [52,53]. We have shown that, although adiponectin is primarily produced by adipocytes, its expression is detectable, and strongly up-regulated by PPAR-γ ligand in normal dermal fibroblasts [11]. Significantly, both RNAi-mediated adiponectin knockdown in normal fibroblasts and genetic depletion of adiponectin in mouse fibroblasts was associated with increased collagen and α-SMA gene expression. Moreover, adiponectin-depleted fibroblasts were sensitized to the profibrogenic effects of TGF-ß. These *in vitro *findings are concordant with *in vivo *observations that adiponectin-null mice developed exaggerated liver fibrosis when challenged with thioacetamide [54,55]. Moreover, adiponectin-deficient hepatic stellate cells failed to respond to the PPAR-γ ligand troglitazone *in vitro*. Together with these observations, our present results indicate that adiponectin plays an important homeostatic role in negative regulation of collagen deposition and myofibroblast accumulation, and that the anti-fibrotic effects associated with endogenous and pharmacological ligands of PPAR-γ are due, at least in part, to activation of the adiponectin/AMP kinase signaling pathway as illustrated in Figure 9. In addition, because scleroderma is associated with impaired PPAR-γ activity [11,24,25], reduced adiponectin levels in scleroderma patients are likely to result from impaired PPAR-γ activity. Considered in the light of these findings, hypo adiponectin coupled with reduced AdipoR1 expression in scleroderma patients with early diffuse disease suggest that attenuated adiponectin signaling is a risk factor directly contributing to the failure to control aberrant fibrogenic responses, resulting in persistent fibroblast activation and sustained collagen production and myofibroblast differentiation. Indeed, low adiponectin levels and reduced adiponectin receptor expression are associated with accelerated development of liver fibrosis in patients with chronic hepatitis C infection [56]. Moreover, low adiponectin or AdipoR1 have been shown to predict progression of hepatic steatosis to cirrhosis [57].

The inverse correlation between adiponectin signaling and fibrogenesis in scleroderma in the aforementioned studies suggests a potential role for adiponectin in the pathogenesis of skin fibrosis. We are struck by the parallels between reduced adiponectin and disappearance of fat tissue in liver fibrosis on the one hand, where quiescent fat-strong hepatic stellate cells transition into fibrogenic myofibroblasts with down-regulation of PPAR-γ, and loss of subcutaneous adipose tissue associated with dermal fibrosis in patients with scleroderma. These parallels raise the intriguing possibility that subcutaneous adipocytes fulfill a role for analogues to that of the hepatic stellate cells of the skin.

## Conclusions

Pharmacological activation of the adiponectin pathway has potent anti-fibrotic effects in normal and scleroderma fibroblasts, and represents an exciting potential therapeutic approach to the control of dermal fibrosis in scleroderma.

## Abbreviations

AdipoR: adiponectin receptor; AICAR: 5-amino-1-β-D-ribofuranosyl-imidazole-4-carboxamide; α-SMA: α-smooth muscle actin; BSA: bovine serum albumin; (D)MEM: (Dulbecco's) modified Eagle's medium; ECM: extracellular matrix; FBS: fetal bovine serum; EGF: epidermal growth factor; LPS: lipopolysaccharide; PGJ_2_: prostaglandin J_2_; PPAR-γ: peroxisome proliferator activated receptor gamma; qPCR: quantitative polymerase chain reaction; siRNA: small interfering RNA;TGF-β: transforming growth factor-ß; TLR4: Toll-like receptor 4.

## Competing interests

The authors declare that they have no competing interests.

## Authors' contributions

FF carried out data analysis and interpretation, and preparation of the manuscript. LL and SB carried out real-time qPCR. YY carried out immunostaining. ZT carried out raft experiments. JW and RGM carried out tissue culture experiments. RS participated in the design of the study. BY participated in coordination and manuscript preparation. JV was involved in study conception and design, manuscript preparation and final approval. All authors read and approved the final manuscript.
